# Sinapic Acid Alleviated Inflammation-Induced Intestinal Epithelial Barrier Dysfunction in Lipopolysaccharide- (LPS-) Treated Caco-2 Cells

**DOI:** 10.1155/2021/5514075

**Published:** 2021-09-08

**Authors:** Huan Lan, Lu-Ying Zhang, Wen He, Wan-Ying Li, Zhen Zeng, Bo Qian, Chengqiang Wang, Jia-Le Song

**Affiliations:** ^1^Department of Analytical Chemistry and Drug Analysis, School of Pharmacy, Guilin Medical University, Guilin, 541004 Guangxi, China; ^2^Department of Nutrition and Food Hygiene, School of Public Health, Guilin Medical University, Guilin, 541004 Guangxi, China; ^3^Department of Maternal and Child Health, XianYa School of Public Health, Central South University, Changsha, 410078 Hunan, China; ^4^State Key Laboratory of Molecular Vaccinology and Molecular Diagnostics, School of Public Health, Xiamen University, Xiamen 361102, China; ^5^Department of Clinical Nutrition, The Second Affiliated Hospital of Guilin Medical University, Guilin 541004, China

## Abstract

The integrity and permeability of the intestinal epithelial barrier are important indicators of intestinal health. Impaired intestinal epithelial barrier function and increased intestinal permeability are closely linked to the onset and progression of various intestinal diseases. Sinapic acid (SA) is a phenolic acid that has anti-inflammatory, antihyperglycemic, and antioxidant activities; meanwhile, it is also effective in the protection of inflammatory bowel disease (IBD), but the specific mechanisms remain unclear. Here, we evaluated the anti-inflammatory of SA and investigated its potential therapeutic activity in LPS-induced intestinal epithelial barrier and tight junction (TJ) protein dysfunction. SA improved cell viability; attenuated epithelial permeability; restored the protein and mRNA expression of claudin-1, ZO-1, and occludin; and reversed the redistribution of the ZO-1 and claudin-1 proteins in LPS-treated Caco-2 cells. Moreover, SA reduced the inflammatory response by downregulating the activation of the TLR4/NF-*κ*B pathway and attenuated LPS-induced intestinal barrier dysfunction by decreasing the activation of the MLCK/MLC pathway. This study demonstrated that SA has strong anti-inflammatory activity and can alleviate the occurrence of high intercellular permeability in Caco-2 cells exposed to LPS.

## 1. Introduction

Intestinal epithelial cells are the key components of the epithelial lining. The intact intestinal epithelial maintains the intestinal physical barrier and plays a critical role in the body's defense functions [1]. Changes in intestinal epithelial barrier permeability incite mucosal inflammation leading to intestinal diseases, such as inflammatory bowel disease (IBD), including Crohn's disease (CD) and ulcerative colitis (UC), irritable bowel syndrome (IBS), and colon cancer (CRC) [2–4]. Intestinal epithelial barriers are formed by tight junction (TJ) proteins, including occludin, claudin, and zonula occludens (ZO), that connect the cytoskeleton and signalling molecules [5]. Intestinal inflammation has been proved to be associated with the intestinal epithelial barrier disruption [6]. Inflammatory stimulation and other endogenous cytokines directly affect the intestinal epithelial barrier by reducing the localization and expression of TJ proteins [7]. Previous studies have indicated that maintenance of the intestinal epithelial barrier and attenuated inflammatory responses is an efficient strategy for the treatment of IBD [8–11].

Lipopolysaccharide (LPS), an important risk factor of inflammation, is widely used in many researches about intestinal tight junction barriers. Stimulation of intestinal cells by LPS induces increased Toll-like receptor-4 (TLR4) expression and triggers the release of proinflammatory mediators [12–14]. What is more, studies suggest that the increase in intestinal epithelial TJ permeability is mediated by the upregulation of myosin light chain kinase (MLCK) by the TLR4/MyD88 and NF-*κ*B signalling pathway [15, 16].

Numerous phenolic compounds of plant origin have been shown to alleviate inflammation and improve intestinal permeability due to their anti-inflammatory and antioxidant ability [17–21]. SA is a well-known phenolic acid that is found in various herbal materials, fruits, and grains, as well as in some vegetables [22, 23]. Modern pharmacological studies have reported that the SA possesses several pharmacological properties including antioxidant [24], anti-inflammatory [22, 25], antihyperglycemic, hypoglycemic [26], and anticancer activities [27]. Although SA has a potential protective role in colitis mice [25], its role in intestinal barrier remains unclear. In view of the important role of TJ proteins in the intestinal epithelial barrier [28], it is necessary for us to investigate the effect of SA on TJ proteins as well as intestinal epithelial barrier. Collectively, this study is aimed at investigating the effects of SA treatment on anti-inflammation and the localization and expression of TJ in an LPS-induced Caco-2 model of inflammation-mediated barrier dysfunction.

## 2. Materials and Methods

### 2.1. Materials and Reagents

Caco-2 cells (purchased from China National Collection of Authenticated Cell Cultures, SCSP-5027) were a gift obtained from Professor Xian-qiong Zou, School of Biotechnology, Guilin Medical University. Sinapic acid (SA) was obtained from Aladdin Biochemical Technology Co., Ltd. (Shanghai, China). Nonessential amino acid (NEAA) solution and LPS were obtained from Solarbio Life Sciences Co., Ltd. (Beijing, China). TRNzol-A+ reagent, FastQuant RT Kit, and SuperReal PreMix Plus (SYBR Green) reagent were obtained from Tiangen Biotech Co., Ltd. (Beijing, China). Triton X-100, fix solution (4% paraformaldehyde), bovine serum albumin (BSA), goat serum (C0265), and other reagents were obtained from Beyotime Biotechnology Co., Ltd. (Shanghai, China). Primary antibodies against ZO-1 (AF8394), occludin (AF7644), claudin-1 (AF6504), NF-*κ*B (AF7569), phospho-NF-*κ*Bp65 (AN371), phospho-I*κ*B*α* (Ser32) (AF5851), phospho-IKK*α*/*β* (Ser176/180) (AI139), MyD88 (AF2116), TLR4 (AF8187), and *β*-actin (AF0003) were purchased from Beyotime Biotechnology Co., Ltd.

### 2.2. Cell Culture and Treatment

Caco-2 cells were routinely cultured at 37°C in in a humidified chamber of 5% CO_2_ in high glucose Dulbecco's Modified Eagle's Medium (DMEM) containing 10% fetal bovine serum (FBS), 1% NEAA, and 1% penicillin/streptomycin. Then, the cells were subcultured at 80–90% confluence. In all experiments, Caco-2 cells were coincubated with LPS (10 *μ*g/ml) in the presence or absence of SA (5, 10, or 15 *μ*mol/l) and incubated for 24 h.

### 2.3. Cell Viability Assay

The MTT assay was used to evaluate the cell viability of SA. The Caco-2 cells were incubated with SA (5, 10, or 15 *μ*mol/l) for 24 h or 48 h. After that, MTT solution (5.0 mg/ml) was added for a further 4 h. The absorbance of the MTT-formazan product was read at 490 nm after dissolving with 150 *μ*l dimethyl sulfoxide (DMSO)/well; each sample was analyzed in quintuplicate (*n* = 3).

### 2.4. Epithelial Permeability Assay

The TJ permeability assays used in this study were the transepithelial electrical resistance (TEER) assay and the fluorescein isothiocyanate- (FITC-) conjugated dextran probe (FD-40) assay. For the TEER and FD-40 assays, monolayer Caco-2 cells were seeded and cultured on 12-well inserts (pore size 0.4 *μ*m; Millipore, Bedford, MA, USA). Then, the Caco-2 cells were treated according to cell culture and treatment groups. The TEER value was determined using an ERS-2 voltohmmeter (Millipore) according to the manufacturer's instructions. For the FD-40 assay, the Caco-2 cells were washed with HBSS (pH 7.4) three times, and FD-40 (1 mg/ml) was subsequently applied to the apical side. After incubation for 2 h at 37°C, the media (100 *μ*l) were collected from the apical to basolateral side, and the fluorescent absorbance was measured by a with ex/em 480/530 nm.

### 2.5. Assessment of NF-*κ*B Nuclear Translocation

Caco-2 cells were seeded in 8-well cover slips (Thermo Fisher, USA). Then, the Caco-2 cells were treated according to cell culture and treatment groups. The slides were fixed and blocked (with 10% goat serum) at 25°C. The first antibodies NF-*κ*Bp65 were used to incubate the slides (overnight at 4°C). After being washed with PBS 3 times, the slides were incubated with fluorescent secondary antibody at 25°C for 1 h. Following PBS washing 3 times, the incubated Caco-2 cells were stained with 4′,6-diamidino-2-phenylindole (DAPI) solution to visualize nuclei. Finally, all images were obtained using a microscope (Leica Microsystems Inc., Buffalo Grove, IL, USA). Cellular characteristics were observed by a microscope with a magnification (50x).

### 2.6. Quantitative Reverse-Transcription- (qRT-) PCR Assay

After different treatment, total RNA was extracted from the Caco-2 cells using a TRIzol-A+ reagent. Extracted RNA reverse transcribed into cDNA according to the kit protocols (Tiangen, Beijing, China). qRT-PCR analysis reacted using QuantStudio™ 6 Flex Real-Time PCR System (Life Technologies, USA) with conditions set to 95°C for 3 min for the initial denaturation and followed by 35~50 cycles with denaturation at 95°C for 10 s, annealing at 56°C for 10 s, and extension at 72°C for 60 s. Based on the 2^-*ΔΔ*^ Ct formula, we calculated relative mRNA levels. The sequences of primer are listed in Table 1.

### 2.7. Protein Extraction and Western Blot Analysis

Caco-2 cells were seeded in 8-well cover slips (Thermo Fisher, USA). Then, the cells were treated according to cell culture and treatment groups. Total proteins from Caco-2 cells were extracted after different treatments using cell lysis buffer for western blotting and IP reagent (Beyotime). The concentration of the extracted proteins was determined using the BCA assay, and the samples were denatured by boiling (100°C) for further study. The total protein samples (30 *μ*g) were separated by SDS-PAGE gel for 90-120 min and then transferred to a PVDF membranes (Millipore). The membrane with separated proteins was blocked with nonfat milk (5%) for 2 h and immediately incubated with first antibodies (overnight at 4°C) *TLR4*, *IKKα*, *p-IKKα*, *MyD88*, *p-NF-κBp65*, *IκB*, and *p-IκB*. After washed with 3 times of PBS, all membranes were incubated with the accordingly secondary antibody for 60 min at 25°C. The blots were visualized with ECL detection reagents (7sea Biotech, Shanghai, China), and the ImageJ software (NIH, Bethesda, MD, America) was employed to numeralization for band analysis (https://imagej.nih.gov/ij/).

### 2.8. Immunofluorescent Localization of TJ Proteins

Localization of the TJ proteins (ZO-1 and claudin-1) was analyzed with immunofluorescence staining. For immunofluorescence staining, the non-SA-treated and SA-treated Caco-2 cells were fixed with fix solution for 15 min and then infiltrated with Triton X-100 for 15 min. Then, the cells were washed and blocked for 1 h with 5% BSA in PBS. After 1 h of blocking with 5% BSA, the cells were incubated with first antibodies (claudin-1 and ZO-1) overnight at 4°C. All cells were then incubated with the corresponding secondary antibodies and stained with DAPI solution for 3 min at room temperature. Images were obtained using a microscope (Leica Microsystems Inc., Buffalo Grove, IL, USA). Cellular characteristics were observed by a microscope with a magnification (100x).

### 2.9. Statistical Analysis

The mean ± standard deviation (SD) is used to describe data. The SPSS 25.0 software (SPSS Inc., Chicago, USA) was employed. One-way ANOVA and Duncan's multiple range tests were used to determine statistically significant differences between the treatments (*p* < 0.05). The GraphPad Prism version 5.0 statistical software package was used for the analysis.

## 3. Results

### 3.1. Toxicity Assay in SA-Treated Caco-2 Cells

**Figure 1:**
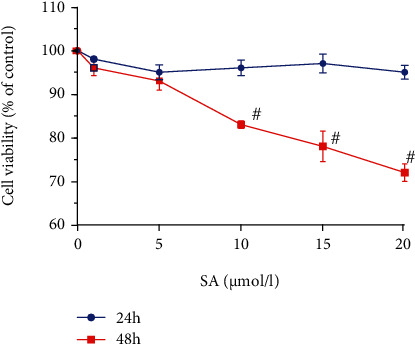
Effect of sinapic acid (SA) on the viability of Caco-2 cells. The viability of Caco-2 cells was determined after treatment with SA (1, 5, 10, 15, or 20 *μ*mol/l) for 24 h and 48 h. The results are expressed as the mean ± SD of three independent experiments. ^#^ denotes *p* < 0.05 vs. the non-SA-treated cells.

The cell viability after 24 h and 48 h of SA treatment (ranging from 1 *μ*mol/l to 20 *μ*mol/l) is shown in Figure 1. SA treatment for 24 h showed no significant effect on cell viability at any concentration compared to the control conditions. Besides, the cell viability in Caco-2 cells was significantly inhibited by SA treatment for 48 h, which effect was in a concentration-dependent manner (*p* < 0.05). A high concentration of SA (20 *μ*mol/l) showed cytotoxicity in Caco-2 cells after 48 h of treatment. Therefore, we selected 5, 10, and 15 *μ*mol/l SA as safe concentration for further study in Caco-2 cells.

### 3.2. Effects of SA on Epithelial Permeability in LPS-Treated Caco-2 Cells

**Figure 2:**
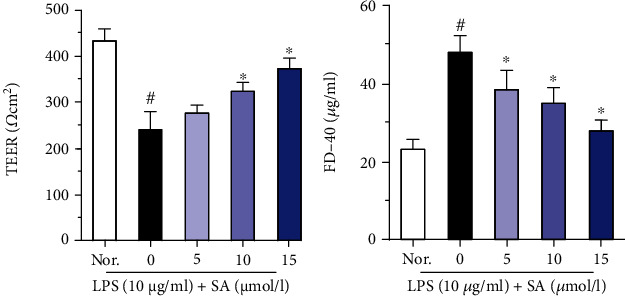
Effects of sinapic acid (SA) on the TEER and FD-40 levels in LPS-treated Caco-2 cells. The TEER and FD-40 levels were determined after treatment with SA (5, 10, or 15 *μ*mol/l) for 24 h in LPS-treated Caco-2 cells. The results are expressed as the mean ± SD of three independent experiments. ^#^ denotes *p* < 0.05 vs. the normal control group (Nor.), and ^∗^ denotes *p* < 0.05 vs. the LPS group.

As shown in Figure 2, LPS treatment induced a significant decrease in TEER values in Caco-2 cells (*p* < 0.05). Administration of SA effectively increased the TEER values in LPS-treated Caco-2 cells. However, SA concentration of 10 and 15 *μ*mol/l led to significantly higher TEER values in Caco-2 cells than the low concentration of SA (5 *μ*mol/l) (*p* < 0.05). In addition, SA induced a concentration-dependent decrease in FD-40 permeability in LPS-treated Caco-2 cells.

### 3.3. Effects of SA on NF-*κ*B Nuclear Translocation in LPS-Treated Caco-2 Cells

**Figure 3:**
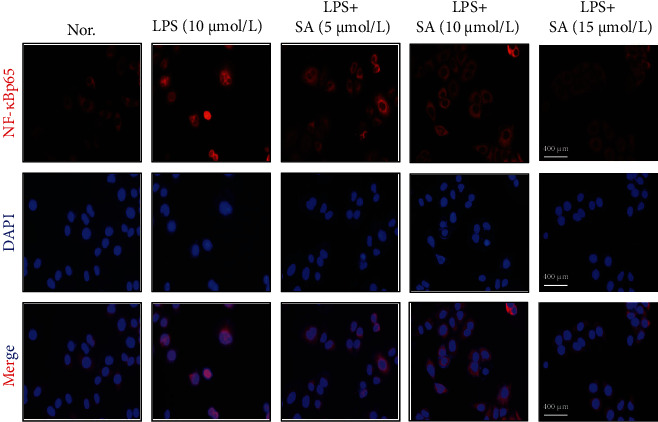
Effect of sinapic acid (SA) on NF-*κ*Bp65 translocation in LPS-treated Caco-2 cells. Caco-2 cells were incubated with LPS (10 *μ*g/ml) and SA (5, 10, or 15 *μ*mol/l) for 24 h and then subjected to immunofluorescence analysis (50x).

As shown in Figure 3, NF-*κ*Bp65 was present in the cytoplasm of untreated Caco-2 cells. LPS treatment markedly increased the activation of nuclear NF-*κ*Bp65 and promoted its translocation (*p* < 0.05). The SA-treated group showed significantly lower translocation of nuclear NF-*κ*Bp65 than that in the LPS group (NF-*κ*Bp65 nuclear translocation rate: 45.45%), and the effect was most significant in the group treated with 15 *μ*mol/l SA (NF-*κ*Bp65 nuclear translocation rate: 18.18%).

### 3.4. Effects of SA on the mRNA Levels of Tlr4, Nf*κ*bp65, Il1*β*, and Il8 in LPS-Treated Caco-2 Cells

**Figure 4:**
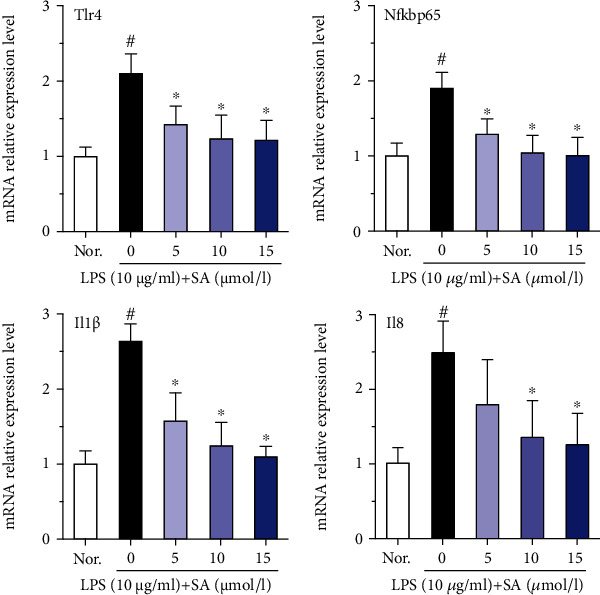
Effects of sinapic acid (SA) on the mRNA levels of *Tlr4*, *Nfκbp65*, *Il1β*, and *Il8* in LPS-treated Caco-2 cells. Caco-2 cells were incubated with LPS (10 *μ*g/ml) and SA (5, 10, or 15 *μ*mol/l) for 24 h and then subjected to qRT-PCR analysis. The results are expressed as the mean ± SD of three independent experiments. ^#^ denotes *p* < 0.05 vs. the normal control group (Nor.), and ^∗^ denotes *p* < 0.05 vs. the LPS group.

IL-1*β* and IL-8 are typical inflammatory cytokines that mediate and promote the inflammatory response. The mRNA levels of Tlr4, Nf*κ*bp65, Il1*β*, and Il8 in Caco-2 cells were analyzed by qRT-PCR assay. Treatment with LPS significantly increased the mRNA expression of Tlr4, Nf*κ*bp65, Il1*β*, and Il8 compared with no treatment (*p* < 0.05; Figure 4). However, this effect was mitigated by treatment with different concentrations of SA (*p* < 0.05). This finding suggests that SA can inhibit the activation of the inflammatory cascade and may decrease the mRNA levels of TLR4/NF-*κ*B signalling pathway components in LPS-treated Caco-2 cells.

### 3.5. Effects of SA on the Protein Levels of TLR4, MyD88, p-NF-*κ*B, p-IKK*α*, and p-I*κ*B in LPS-Treated Caco-2 Cells

**Figure 5:**
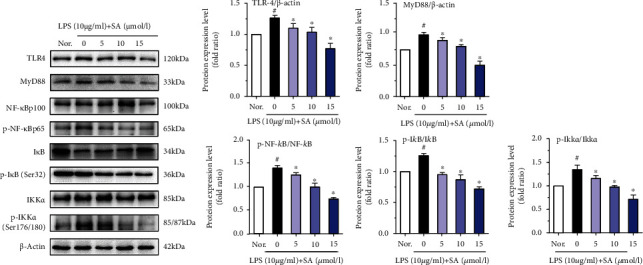
The effects of sinapic acid (SA) on the protein levels of NF-*κ*B-related factors in LPS-treated Caco-2 cells. Caco-2 cells were incubated with LPS (10 *μ*g/ml) and SA (5, 10, or 15 *μ*mol/l) for 24 h and then subjected to western blot analysis. The results are expressed as the protein expression level (normalized to *β*-actin) relative to that in unstimulated cells and are shown as the mean ± SD of three independent experiments. ^#^ denotes *p* < 0.05 vs. the normal control group (Nor.), and ^∗^ denotes *p* < 0.05 vs. the LPS group.

The protein expression levels of TLR4, MyD88, p-NF-*κ*Bp65, p-IKK*α*, and p-I*κ*B were analyzed using a protein blotting assay, and the effect of SA on the TLR4/NF-*κ*B pathway was evaluated. As shown in Figure 5, the protein levels of MyD88, p-NF-*κ*Bp65, TLR4, p-IKK*α*, and p-I*κ*B were increased following treatment with LPS (*p* < 0.05). However, administration of different concentrations of SA modulated the expression levels of these proteins (*p* < 0.05). These results clearly indicate that SA inhibits the TLR4/NF-*κ*B signalling pathway in LPS-treated Caco-2 cells.

### 3.6. Effects of SA on the Protein Levels of MLCK and MLC in LPS-Treated Caco-2 Cells

**Figure 6:**
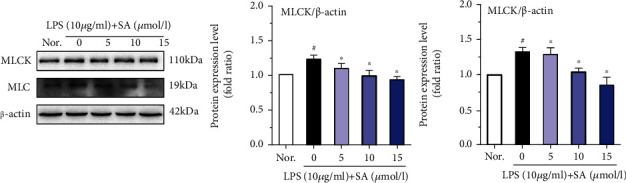
The effects of sinapic acid (SA) on the protein levels of MLCK and MLC in LPS-treated Caco-2 cells. Caco-2 cells were incubated with LPS (10 *μ*g/ml) and SA (5, 10, or 15 *μ*mol/l) for 24 h and then used for western blot analysis. The results are expressed as the protein expression level (normalized to *β*-actin) relative to that in unstimulated cells and are shown as the mean ± SD of three independent experiments. ^#^ denotes *p* < 0.05 vs. the normal control group (Nor.), and ^∗^ denotes *p* < 0.05 vs. the LPS group.

MLCK regulates the spatial conformation and function of the cytoskeleton and TJ of intestinal epithelial barrier cells. LPS treatment significantly induced the activation of MLCK and MLC (*p* < 0.05; Figure 6). In addition, treatment with different concentrations of SA significantly reduced the activation of MLCK and MLC in LPS-treated Caco-2 cells (*p* < 0.05).

### 3.7. Effects of SA on the mRNA Levels of ZO-1, Claudin-1, and Occludin in LPS-Treated Caco-2 Cells

**Figure 7:**
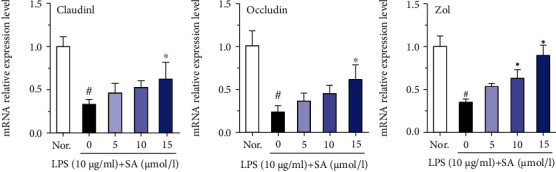
Effects of sinapic acid (SA) on tight junction mRNA levels in LPS-treated Caco-2 cells. Caco-2 cells were incubated with LPS (10 *μ*g/ml) and SA (5, 10, or 15 *μ*mol/l) for 24 h and then subjected to qRT-PCR analysis. The results are expressed as the mean ± SD of three independent experiments. ^#^ denotes *p* < 0.05 vs. the normal control group (Nor.), and ^∗^ denotes *p* < 0.05 vs. the LPS group.

Normal levels of ZO-1, claudin-1, and occludin, the main members of the TJ protein family, act as a vital role in maintaining normal intestinal barrier function. qRT-PCR analysis showed that LPS treatment significant decreases in the mRNA levels of Zo1, claudin-1, and occludin in Caco-2 cells (Figure 7). However, administration of SA increased the mRNA levels of Zo1, claudin-1, and occludin in LPS-treated Caco-2 cells. At a concentration of 15 *μ*mol/l, SA markedly increased the mRNA expression of these TJ factors (including Zo1, claudin-1, and occludin) (*p* < 0.05).

### 3.8. Effects of SA on the Protein Levels of Occludin, Claudin-1, and ZO-1 in LPS-Treated Caco-2 Cells

**Figure 8:**
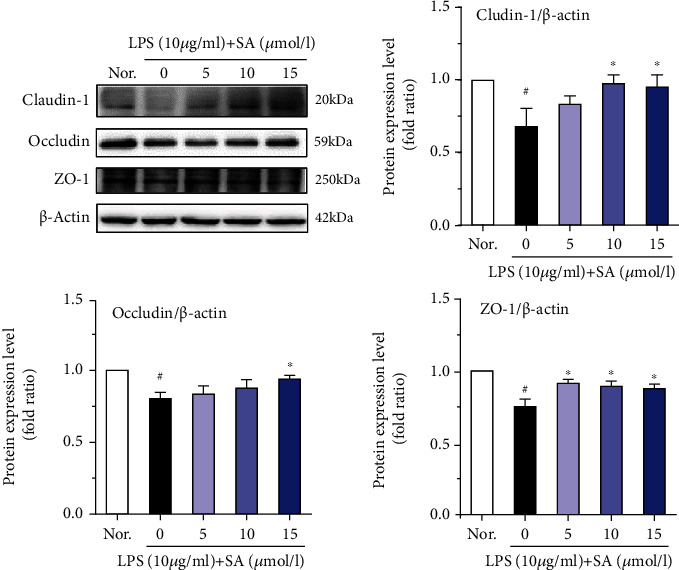
Effects of sinapic acid (SA) on tight junction protein levels in LPS-treated Caco-2 cells. Caco-2 cells were incubated with LPS (10 *μ*g/ml) and SA (5, 10, or 15 *μ*mol/l) for 24 h and then used for western blot analysis. The results are expressed as the protein expression level (normalized to *β*-actin) relative to that in unstimulated cells and are shown as the mean ± SD of three independent experiments. ^#^ denotes *p* < 0.05 vs. the normal control group (Nor.), and ^∗^ denotes *p* < 0.05 vs. the LPS group.

LPS reduced the protein levels of ZO-1, claudin-1, and occludin in Caco-2 cells (*p* < 0.05; Figure 8). However, treatment with SA (15 *μ*mol/l) markedly attenuated the downregulation of these TJ proteins (*p* < 0.05).

### 3.9. Effects of SA on Claudin-1 and ZO-1 Localization and Distribution in LPS-Treated Caco-2 Cells

**Figure 9:**
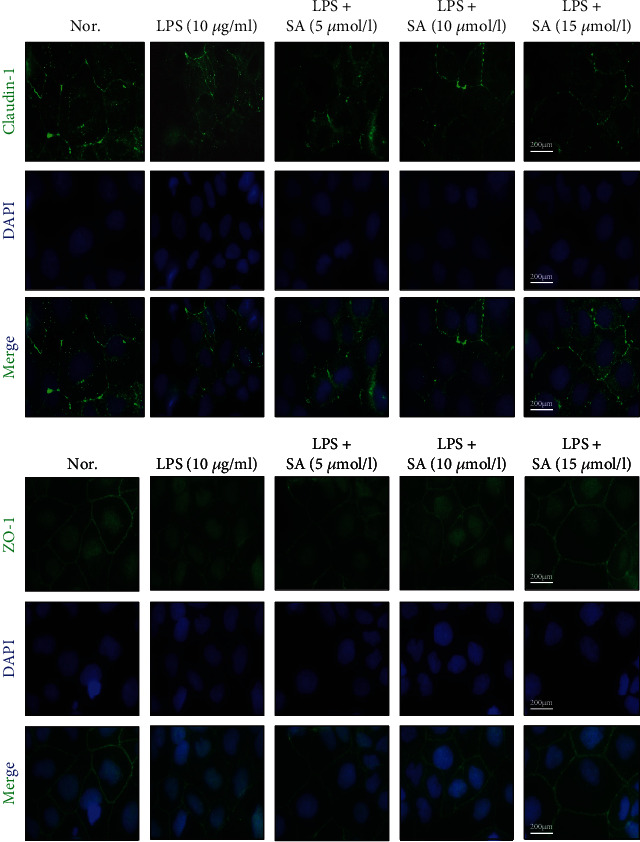
Effects of sinapic acid (SA) on claudin-1 and ZO-1 localization and distribution in LPS-treated Caco-2 cells. Caco-2 cells were incubated with LPS (10 *μ*g/ml) and SA (5, 10, or 15 *μ*mol/l) for 24 h and then subjected to immunofluorescence analysis (100x).

As shown in Figure 9, claudin-1 and ZO-1 were appropriately localized to their respective intercellular junctions and were connected without damage in untreated Caco-2 cells. However, local claudin-1 and ZO-1 staining in the pericellular was discontinuous in the cells treated with LPS. These discontinuous pericellular expressions of both claudin-1 and ZO-1 were counteracted by SA treatment, and a strong fluorescence intensity was observed at the periphery of the cells.

## 4. Discussion

Plant-derived phenolic compounds are a type of organic acid that contains either a benzoic or cinnamic acid skeleton with phenol as the basic framework and a relatively simple structure. Recent studies have suggested that SA has anti-inflammatory effects on intestinal inflammation and can regulate the intestinal microbiota and improve the redox state [25, 29, 30]. In the current investigation, we attempted to appraise the effects of SA on intestinal inflammation and permeability. Our results clearly confirmed that SA reduced epithelial permeability, increased the expression of the TJ proteins, and attenuated LPS-induced inflammation by modulating the TLR4/NF-*κ*B pathway.

The intestinal epithelial barrier can effectively prevent pathogenic microorganisms, antigens, and toxic substances from entering the body from the gut [31, 32]. Epithelial barrier function is mediated by intercellular junctions [33]. TJ formation and assembly involve a complex of proteins. The TJ proteins (such as ZO-1, occludin, and claudin-1) are generally known as the main event during the change course of intestinal permeability [34]. Therefore, our investigation focused on the expression and distribution of ZO-1, occludin, and claudin-1.

Numerous studies have shown that LPS increases intestinal barrier permeability regulates the expression of TJ proteins and eventually lead to intestinal barrier dysfunction [35, 36]. On the other hand, the excessive accumulation of proinflammatory cytokines, including IL-1*β* and IL-8, is associated with promoting the generation of inflammation and ultimately causes the destruction of the intestinal epithelial barrier. LPS decreases TJ proteins in intestinal act as a centrical role in the cellular mechanisms of intestinal barrier defects [32]. In our study, LPS (10 *μ*g/ml) was used to successfully establish the inflammation model. Here, our results suggested that the LPS-induced increases in the IL-1*β* and IL-8 expression and decreases in the protein levels of ZO-1, occludin, and claudin-1 were alleviated by SA.

TLR4, one of the best characterized pattern recognition receptors, is activated by LPS, leading to the activation of NF-*κ*B and subsequently inducing the production of proinflammatory mediators. Recently, many studies have indicated that NF-*κ*B is a key factor in inflammatory gene expression [37–39]. Under normal conditions, NF-*κ*B binds with I*κ*B to form an inactivation complex. NF-*κ*B is released through I*κ*B kinase (IKK), leading to I*κ*B-*α* phosphorylation and degradation. Some stimulatory factors, such as proinflammatory cytokines, antigen receptors, growth factors, and LPS, may activate the I*κ*B kinase (IKK) complex, which phosphorylates I*κ*B. The phosphorylation of I*κ*B causes its ubiquitination and proteasomal degradation, releasing NF-*κ*B from the complex. In addition, NF-*κ*B subunit p65 is transferred from the cytoplasm into the nucleus and initiates an inflammatory response [40]. A study reported that cinnamic acid downregulated the protein and mRNA expression of p-NF-*κ*B and p-IKK*α*/*β*, which exerted anti-inflammatory effects in LPS-treated Caco-2 and RAW264.7 coculture systems [41]. Similar results were also found that ferulic acid treatment decreased the phosphorylation of I*κ*B and NF-*κ*Bp65 in LPS-treated bovine endometrial epithelial cells [42]. Here, these results suggested that that SA treatment significantly diminished the activation of NF-*κ*Bp65; decreased the mRNA or protein expression of TLR4, MyD88, p-NF-*κ*B, p-IKK*α*, and p-I*κ*B; and inhibited LPS-induced IL-1*β* and IL-8 expression via modulation of the TLR4/NF-*κ*B pathway. Moreover, the effects of SA treatment were concentration-dependent. These results suggest that SA has strong anti-inflammatory activity and can inhibit the LPS-induced activation of TLR4/NF-*κ*B signalling pathway.

Activation of the MLCK-MLC pathway mainly regulates the spatial conformation and function of the cytoskeleton and plays a key role in regulating the tight junctions of intestinal cells. MLCK is a Ca^2+^/calmodulin-dependent kinase. Under physiological or pathological conditions, the tight junctions of cells can be regulated, and the cytoskeleton can be remodeled by catalyzing the phosphorylation of MLC, thus affecting the permeability of the intestinal epithelium [43]. It has been reported that overexpression of MLCK leads to increase intestinal epithelial TJ permeability [15, 44]. Many studies have reported that increased MLCK activity phosphorylates MLC and subsequently leads to the centripetal contraction of the TJ complex, eventually result in the opening of the intestinal epithelial barrier [45–47]. The results from our study clearly indicate that LPS promoted the expression of MLCK and MLC and diminished the expression of key sealing TJ proteins, such as ZO-1, occludin, and claudin-1, causing varying degrees of displacement of the claudin-1 and ZO-1 proteins. In contrast, SA inhibited the overactivation of MLCK and partially restored the expression and localization of related TJ proteins, reflecting that SA may alleviate injury to the intestinal epithelial barrier by inhibiting the MLCK/MLC pathway.

## 5. Conclusions

This study demonstrated that SA could ameliorate damage to the intestinal epithelial barrier and inhibit inflammation in LPS-stimulated intestinal epithelial cells. SA restored tight junction protein expression and protein localization via inhibition of the TLR4/NF-*κ*B/MLCK-MLC pathway. Future research will reveal in more detail the potential of SA in the treatment or prevention of inflammation-induced intestinal barrier defects. In addition, SA also has the potential to be a possible alternative for the treatment of gastrointestinal diseases.

## Figures and Tables

**Table 1:** Primers used to measure mRNA expression.

Gene	Forward	Reverse
*Gapdh*	CCATTTGATGTTAGCGGGATCTC	TGGTCTACATGTTCCAGTATGACT
*Tlr4*	GTACCTGGGGAACAACCTCTT	GCAGCTTGACTAGACTCTCCA
*Nfκbp65*	GTGGGGACTACGACCTGAATG	GGGGCACGATTGTCAAAGATG
*Il1β*	GAATGACGCCCTCAATCAAAGT	TCATCTTGGGCAGTCACATACA
*Il8*	CCTGAACCTTCCAAAGATGGC	TTCACCAGGCAAGTCTCCTCA
*Mlck*	CAACAGGGTCACCAACCAGC	GCCTTGCAGGTGTACTTGGC
*Occludin*	CTTCCAATGGCAAAGTGAATG	TACCACCGCTGCTGTAACGAG
*Claudin-1*	CCAGGTACGAATTTGGTCAGG	TGGTGTTGGGTAAGAGGTTGT
*Zo1*	GAGCCTAATCTGACCTATGAACC	TGAGGACTCGTATCTGTATGTGG

## Data Availability

All generated and analyzed data used to support the findings of this study are included within the article.
